# Sexual communication in castniid moths: Males mark their territories and appear to bear all chemical burden

**DOI:** 10.1371/journal.pone.0171166

**Published:** 2017-02-08

**Authors:** Carmen Quero, Victor Sarto i Monteys, Gloria Rosell, Marc Puigmartí, Angel Guerrero

**Affiliations:** 1 Department of Biological Chemistry and Molecular Modelling, IQAC (CSIC), Barcelona, Spain; 2 Institute of Environmental Science and Technology (ICTA), Entomology, Plants and Health. ICTA-ICP Building, Autonomous University of Barcelona, Bellaterra (Barcelona), Spain; 3 Department of Agriculture, Livestock, Fisheries and Food (DARP), Catalonian Government—Service of Plant Health, Barcelona, Spain; 4 Department of Pharmacology and Therapeutic Chemistry (Unit Associated to CSIC), University of Barcelona, Barcelona, Spain; Montana State University Bozeman, UNITED STATES

## Abstract

Castniid moths (Lepidoptera: Castniidae) display a butterfly-like reproductive behavior, i.e., they use visual stimuli for mate location and females have apparently lost their pheromone glands in an evolutionary context. In this paper we report for the first time the identification of three new compounds, namely *n*-octadecyl acetate, (Z)-9-octadecenyl acetate and (E,Z)-2,13-octadecadienyl acetate, in males of the Castniid Palm Borer, *Paysandisia archon*, which could be involved in its short-range courtship behavior, and also shed light on recent controversies on the sexual behavior of the species. The compounds are produced in a ring-shaped gland of the male terminalia and have occasionally been detected in very minor amounts (ng) in ovipositor extracts of females, but only while mating or just after copulation. We also report that males use the already known (E,Z)-2,13-octadecadienol to mark their territory by rubbing their midlegs against the upper side of nearby leaves, especially palm leaves. This compound, produced in large amounts, is mostly concentrated in the midleg basitarsi and its maximum production is detected on the sexually mature 1-day-old specimens. In addition, analysis of male wings extracts confirms the presence of Z,E and E,E-farnesals, which are mostly produced in the median band of hindwings of 48–53 h-old insects. The biological significance of farnesals in this species is unknown. Our results point out that the chemical communication of *P*. *archon* relies mostly on males, which appear to bear all chemical burden in this respect.

## Introduction

Intraspecific chemical communication in insects relies on the release of pheromones that play a crucial role in many behavioral and physiological responses [1]. In Lepidoptera, female moths generally release long range pheromones to attract conspecific mates, and when males are close enough they may release short-range pheromones to help seduce the female, i.e. to facilitate the final steps leading to copula [2]. Although female calling is a general mate-finding strategy in diurnal and nocturnal moths, day-flying butterflies only use visual cues for mate-finding, followed by short-range pheromones released by males when the two sexes are in close proximity [3–5].

The Castniidae is a small family (113 species described) of bright-colored day-flying moths which occurs in the Neotropics, Southeast Asia and Australia [6, 7]. Currently, it is grouped within the superfamily Cossoidea with six families: Brachodidae, Cossidae, Dudgeoneidae, Metarbelidae, Ratardidae, and Sesiidae [7–10]. The Neotropical species of castniids mimic many butterflies coexisting in the same habitat in form, colors and habits [11], an unprecedented case in Lepidoptera between two phylogenetically distant groups [4]. This fact has granted the Neotropical castniids the name “butterfly-moths”, whereas the Australian and Asian species are commonly known as “sun moths”. In addition, castniid males are territorial displaying perching behavior as many butterfly males [11–14]. Castniid females, as butterfly females, appear to have lost their abdominal glands, and therefore they do not release long-range pheromones to attract conspecific males. This was initially hypothesized by Sarto i Monteys and Aguilar [14] and later evidenced in studies carried out on the Castniid Palm Borer *Paysandisia archon* (Burmeister) [12, 13], recently introduced to Europe from South America.

However, against this evidence, Delle-Vedove et al. (2014) [15] claimed that sexually mature *P*. *archon* females release a pheromone to attract males in a moth-butterfly hybrid strategy implying both chemical and visual clues. To clarify this point and to shed light on its sexual behavior, we present our latest findings on the chemical communication of this insect, which could also be useful for the development of a strategy to control this invasive species.

## Materials and methods

### Insects

Live cocoons of *P*. *archon* were collected at the beginning of summer (late June- early July) at Béziers and Saint-Guiraud (Hérault, SE France) in 2013 and 2014 respectively, and at Santa Llogaia del Terri (Girona, NE Spain) in 2015–2016. Cocoons were found hidden in the upper part of trunks of either Canary island date palms *Phoenix canariensis* hort. ex Chabaud (French sites) or Chinese windmill palms *Trachycarpus fortunei* (Hook.) H. Wendl. (Spanish site), and were placed in insectaries (50 × 25 × 32 cm^3^) of the ICTA-Autonomous University of Barcelona facilities at laboratory temperature (22–25°C). Insectaries were checked daily and hourly from 8:00 to 14:00 h to look for adult emergences, which only occur in the morning. After emergence, adults were sexed, tagged for age records, and either placed in semi-field conditions within a 1.20 × 1.50 × 2.10 m^3^ wire mesh cage, located in a nearby forest where sexes could restrictively fly and mate at will, or refrigerated at 18°C in labelled 500 cm^3^ plastic containers.

In summer 2015, a wild adult population of *P*. *archon* was found in a small palm plot (*ca*. 51 m^2^) at Sant Fost de Campsentelles, Barcelona, Spain. It contained eleven palm trees belonging to four palm species (*T*. *fortunei*, European fan palm *Chamaerops humilis* L., California fan palm *Washingtonia filifera* (Lindl.) H.Wendl., and Date palm *Phoenix dactylifera* (L.) Mill.). *P*. *archon* male specimens were photographed while perching (S1 Fig), so that they could be singled out and identified in the next days. Wing and body size combined with wing spots and marks were sufficient to help identify the males in the perching zone. The specimens were monitored undisturbed in this natural habitat for 3–4 days a week for 4 consecutive weeks. While perching, which is done mostly on palm leaves, males may also do rubbing (see below). In three cases (two with one young male, and one with an old male) we were able to single out the palm leaf surfaces on *C*. *humilis*, where these males had been rubbing. Immediately after the rubbing observations, the rubbed leaf area (*ca*. 50–100 cm^2^) was cut with scissors, placed into an airtight plastic bag, labelled and refrigerated in a portable field cooler, and taken to the laboratory in 30–60 min. Each leaf sample was immediately cut further into smaller pieces (5±1 mm^2^), weighed (3.5±1 g) and placed into a glass vial for analysis (see below for details).

### GC-MS analyses

Wings and legs were separated from the body of the insect and cut into pieces (5±1 mm^2^ for the wings and 5–10 mm for the legs) before being immersed in hexane for 1 h. Male terminalia, male glands and female ovipositors were excised under a stereo microscope and immediately submerged in hexane for 1 h. The supernatant was decanted and all extracts were stored at -80°C until use. The volume of hexane used for each extract was adapted to just cover the whole sample (2 mL for wing extracts and 500–750 μL for the other extracts). For the rubbing experiments, *C*. *humilis* leaves, which had been rubbed or not (control) by *P*. *archon* males (see above), were covered with 1 mL of hexane/g of vegetal material, and subjected to the same procedure as for the body extracts. For ovipositor extracts, 6 groups of virgin females, 4 of mated females and 4 of “during-mating” females, each of them containing two ovipositors, were obtained from females of similar age. Then, 10 μL of a 0.5 ng/μL of dodecyl acetate in hexane as internal standard (IS) were added, and the extracts concentrated to 1–2 μL for analysis. Terminalia and male glands were analyzed individually and the two pairs of wings or legs of the same insect were considered for the extracts. Extracts were analyzed by GC-MS in splitless mode on a Finnigan Trace 2000 GC system (Thermo Fisher Scientific) coupled to a Trace MS quadrupole mass spectrometer (Thermo Fisher Sci.) working in electron impact (EI) mode. To 290 μL of extract were added 10 μL of a 60 ng/μL solution of dodecyl acetate (12:Ac) or (Z)-9-tetradecenol (Z9-14:OH) in hexane as IS. Quantification of the amounts of farnesals and (E,Z)-2,13-octadecadienol (E2,Z13-18:OH) was done through a calibration curve with solutions of these chemicals in hexane at concentrations 1, 2.5, 5, 10 and 20 ng/μL.

The GC-MS analyses were implemented using an HP-5MS (30 m, 0.25 mm i.d. x 0.25 μm) capillary column (Agilent Technologies) under the following chromatographic conditions: injection at 60°C for 1 min, program of 10°C/min up to 220°C, followed by 5°C/min up to 280°C which was maintained for 10 min. For Z/E isomeric analyses, a polar DB-wax (30 m, 0.25 mm i.d. × 0.25 mm) capillary column (Supelco) was used under the following conditions: injection at 60°C for 1 min, program of 10°C/min up to 240°C, which was kept for 10 min. Helium (1 mL/min) was used as carrier gas. The mass range was m/z 40–500 with a scan time of 1 s. Identification of compounds was done by comparison of their mass spectra and retention times with those of synthetic or commercial chemicals, and/or from a commercial library (NIST Registry of Mass Spectral Data, 2005).

### Statistical analysis

Prior to statistical analysis, all the data were checked for normality and variance homogeneity (Shapiro–Wilk's and Levene's tests, respectively). When these assumptions were not accomplished, i.e. mean amounts of farnesals in hindwings and forewings and mean amounts of dienol in legs, the data were transformed to log (x+1). Two pairwise comparison by Student’s t-test was made between the mean abundance of the components found in each case (terminalia vs male gland and forewing vs hindwing). One-way analysis of variance (ANOVA) followed by LSD post-hoc test was used to compare the mean amount of each component present in forewings and hindwings between individuals of different ages. All statistical analyses were performed in SPSS v 13.0 (SPSS Inc., Chicago, IL, USA).

### Chemicals

Dimethyl disulphide (DMDS), *n*-octadecyl acetate (18:Ac)and farnesals were purchased from Sigma-Aldrich. (Z)-13-Octadecenyl acetate (Z13-18:Ac) and (E)-13-octadecenyl acetate (E13-18:Ac) had been synthesized previously in our laboratory, (Z)-2-octadecenyl acetate (Z2-18:Ac) and (E)-2-octadecenyl acetate (E2-18:Ac) were obtained by stereoselective reduction of the acetylenic alcohol precursor with hydrogen/Lindlar catalyst and lithium aluminum hydride, respectively. (E)-3-Octadecenyl acetate (E3-18:Ac) was prepared by reduction of the tetrahydropyranyl ether-protected acetylenic precursor with sodium in ammonia. (Z)-9-Octadecenyl acetate (Z9-18:Ac) was obtained commercially from TCI Europe. E2,Z13-18:OH and its acetate (E2,Z13-18:Ac) were kindly provided by Sociedad Española de Desarrollos Químicos (SEDQ, Barberà del Vallès, Barcelona, Spain).

### Derivatization with DMDS

Localization of the double bond(s) in the mono- and diunsaturated compounds of the terminalia extracts was determined by DMDS derivatization [16], followed by assignment of the corresponding fragments of the mono- and bis-adduct, respectively. Briefly, to 100 μL of the terminalia extract were added 100 μL of DMDS and 5 μL of a 60 mg/mL solution of iodine in ether and the mixture was heated at 40°C for 24 h. The mixture was cooled, diluted with 200 μL of hexane, and the iodine was removed by treatment with 100 μL of a 5% aqueous solution of Na_2_S_2_O_3_. The organic phase was concentrated to ca. 10 μL and kept at −80°C until GC-MS analysis.

### Electroantennogram assays

The electroantennogram (EAG) apparatus was commercially available from Syntech (Kirchzarten Germany). In brief, antennae of 5–6 day-old females (1 virgin and 4 mated) of *P*. *archon* were excised, cut on both ends, and fixed to both electrodes with conducting gel Spectra 360 (Parker lab. Inc., Hellendoorn, The Netherlands). A glass tube (7 cm long x 6 mm diameter) with three openings was used as a stimulus dispenser. One of these openings was connected to a continuous flow of humidified pure air, the second allowed a complementary air current to keep a constant air pressure, and the third one, close to the antenna, housed the stimulus source. Test stimulations were carried out by giving series of puffs of air (300 ml/min) for 200 ms through a Pasteur pipette with the aid of a CS-01 stimulus controller (Syntech). The pipette contained a small piece of filter paper (1.5 cm diameter) on which had been deposited 1 and 10 μg of Z9-18:Ac, 18:Ac and E2,Z13-18:Ac diluted in hexane, and a mixture of them in 100:60:40 ratio, respectively. The solvent was allowed to evaporate before the tests. Test compounds were puffed on the antenna at intervals of 60 s. Control puffs through a piece of paper containing only solvent (hexane) were also intercalated between two consecutive stimuli to determine the baseline depolarization of the antennae. The signals were amplified (10x), filtered (DC to 1 kHz) with an IDAC-2 interface (Syntech), further amplified (10x), digitized on a PC, and analyzed with the EAG Pro program.

### Ethics statement

This study refers to a moth that is not an endangered or protected invertebrate species. All necessary permits were obtained for the studies described.

## Results

### Male wings

**Fig 1:**
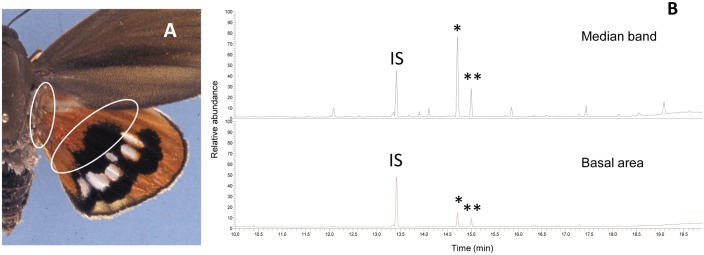
Analysis of extracts of hindwing sections of *Paysandisia archon* males. (A) Hindwing sections (basal area, i.e. near the thorax, and median band) of 7–9 h old *P*. *archon* males. (B) Chromatogram of a GC-MS analysis of extracts of these areas showing the presence of (Z,E)-farnesal (*) and (E,E)-farnesal (**). IS = Z9-14:OH (Photo V. Sarto i Monteys).

Extracts of forewings and hindwings of males of different ages (7–9 h age considered as 0 days old, 20–24 h as 1 day old, and 48–53 h as 2 days old) confirmed the presence of (Z,E)-3,7,11-trimethyl-2,6,10-dodecatrienal ((Z,E)-farnesal) and its E,E isomer ((E,E)-farnesal) [4, 13]. The detected amounts of both isomers in the hindwings were higher than those found in the forewings in all groups of age considered (Student's t-test, P < 0.05), with the amount of (Z,E)-farnesal ranging from 92±14 (males of 20–24 h) to 1194±421 ng/wing (males of 48–53 h) found in the forewings, and from 1104±55 (males of 20–24 h) to 12207±4020 ng/wing (males of 48–53 h) in the hindwings (Table 1). The amounts of either isomer did not apparently increase over the first day of the male but they significantly rose in 2 day-old males both in forewings (Z,E isomer: F_2,16_ = 10.713, P<0.001 and E,E isomer: F_2,16_ = 6.896, P<0.01) and in hindwings (Z,E isomer: F_2,16_ = 6.823, P<0.01 and E,E isomer: F_2,16_ = 9.905, P<0.01). To know more precisely where the farnesals were produced, two sections of the hindwings, the median band and the basal area were extracted and analyzed (Fig 1).

**Table 1:** Mean amount (±SD) of Z,E- and E,E-farnesals detected in the forewings and hindwings of *P*. *archon* males of different ages^1^^,^^2^.

	Z,E			E,E		
	7–9 h^3^	20–24 h^4^	48–53 h^5^	7–9 h^3^	20–24 h^4^	48–53 h^5^
Forewings	115±21a	92±14a	1194±421b	53±11a	40±8a	324±99b
Hindwings	1364±263a	1104±55a	12207±4020b	503±120a	323±27a	2001±487b
Significance^6^	P = 0.002	P = 0.007	P<0.001	P<0.001	P = 0.034	P = 0.013

^1^In ng/wing.

^2^Different letters within the same row indicate significant differences among the mean amounts for each isomer (One-way ANOVA test (P < 0.05) followed by LSD post-hoc test).

^3^(N = 8).

^4^(N = 4).

^5^(N = 7).

^6^P values showing significant differences between the mean amounts in forewings and hindwings of males of the same age (Student's *t*-test, P < 0.05).

The median band contained a mean of 4463±211 ng/hindwing (N = 2) of the Z,E isomer and 1234±268 ng/hindwing (N = 2) of the E,E isomer, whereas the basal area contained only 395±80 and 152±27 ng/hindwing (N = 2) of the two isomers, respectively.

### Male legs

**Fig 2:**
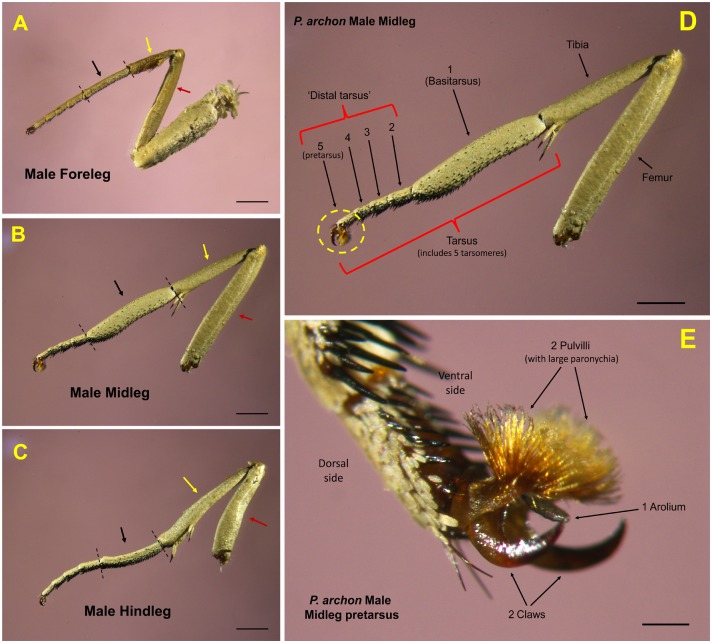
Midleg of a *Paysandisia archon* male. (A) Foreleg, (B) Midleg and (C) Hindleg of the same specimen (scale bar 2 mm). Coxa and trochanter are not shown in midleg and hindleg. Red, yellow and black arrows signal femur, tibia and basitarsus, respectively. In the midlegs the 1^st^ tarsomere or basitarsus (delimited by dashed lines) is notably enlarged with respect to that of forelegs and hindlegs. (D) Side view of full midleg (excluding coxa and trochanter). Terminal circle (yellow dashed line) shows the 5^th^ tarsomere or pretarsus, which holds the paronychia and claws. Scale bar 2 mm. (E) Close-up of pretarsus seen lateroventrally, and showing the two pulvilli with large brush-like paronychia, which seem to be used by males to spread the dienol on palm leaves and nearby plants. Scale bar 0.25 mm (Photos V. Sarto i Monteys).

The dienol E2,Z13-18:OH was already known to be produced in huge amounts in male midlegs [17] but two important points remained unknown: a more precise site of production and/or storage within the midlegs, and quantification of its presence in different midleg segments in males of different ages. Indeed, analysis of new male leg extracts showed the presence of this dienol in large amounts only in the midlegs, being absent in hindlegs and forelegs. The amount of dienol was age-dependent, being highest in males of 20–25 h old (774±432 μg/leg, N = 4) and lowest in males of 7–9 h old (308±276 μg/leg, N = 3) (Table 2). To know more precisely the site of production of the dienol, male midlegs were cut into three sections: tibia, basitarsus and “distal tarsus” (Fig 2).

**Table 2:** Mean amount (±SD) of E2,Z,13–18:OH detected in the tibia, basitarsus and “distal tarsus” of *P*. *archon* males of different ages^1^.

	Tibia	Basitarsus	“distal tarsus”
7–9 h^2^	0.30±0.03	308±276	1.05±0.23
20–25 h^3^	0.34±0.04	774±432	0.81±0.60
48–54 h^2^	0.32^4^	477±230	0.57±0.20

^1^In μg/leg.

^2^(N = 3).

^3^(N = 4).

^4^Detected in only one extract.

**Fig 3:**
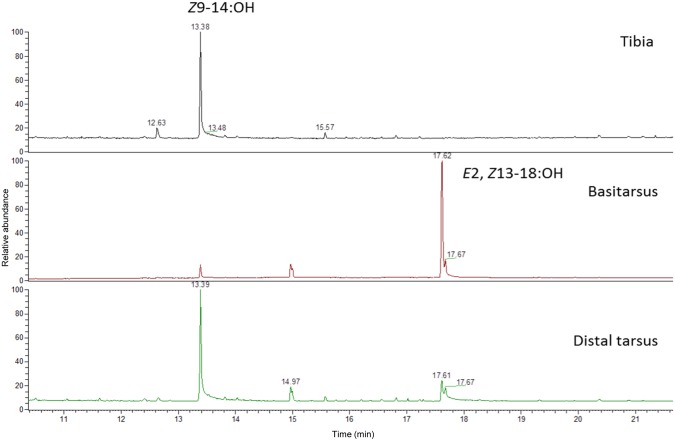
Analysis of extracts of tibia, basitarsus and “distal tarsus” of a *P*. *archon* male. Chromatogram of a GC-MS analysis showing the almost exclusive presence of E2,Z13-18:OH in the basitarsus (Z9-14:OH as IS).

The basitarsi extracts contained the dienol almost exclusively (546±367 μg/basitarsus vs 0.86±0.45 μg/**“**distal tarsus” and 0.36±0.04 μg/tibia) (One-way ANOVA test (P < 0.001), followed by LSD post-hoc test, F_2,23_ = 28.42, P<0.001) (Fig 3). Here, again, the mean amount of dienol present in the basitarsi was also dependent on male age being highest in 20–25 h-old males (Table 2). The amount of compound in the tibia and “distal tarsus” was much lower in males of all ages considered.

### Male terminalia

**Fig 4:**
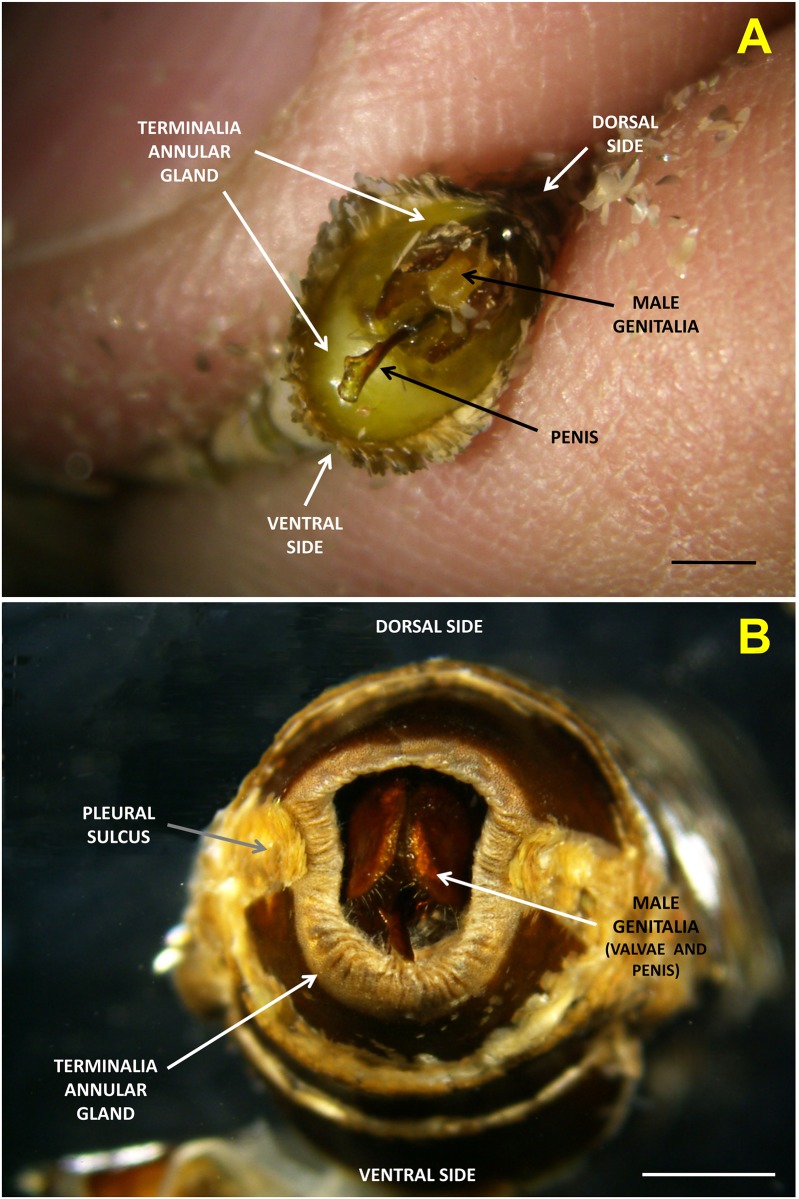
Terminalia annular gland of a *Paysandisia archon* male. (A) Terminalia of a live *P*. *archon* male showing the expanded ring-shaped gland that produces the three acetates Z9-18:Ac, E2,Z13-18:Ac and 18:Ac mentioned in the text. Scale bar 2 mm. (B) Terminalia of a dead *P*. *archon* male (preserved in 70% ethanol) with all terminal scales removed (except the pleural scales) showing the ring-shaped gland. The epithelial gland appears shrinked but numerous folds can be seen allowing for its expansion (as seen in A). Scale bar 2 mm. (Photos V. Sarto i Monteys).

**Fig 5:**
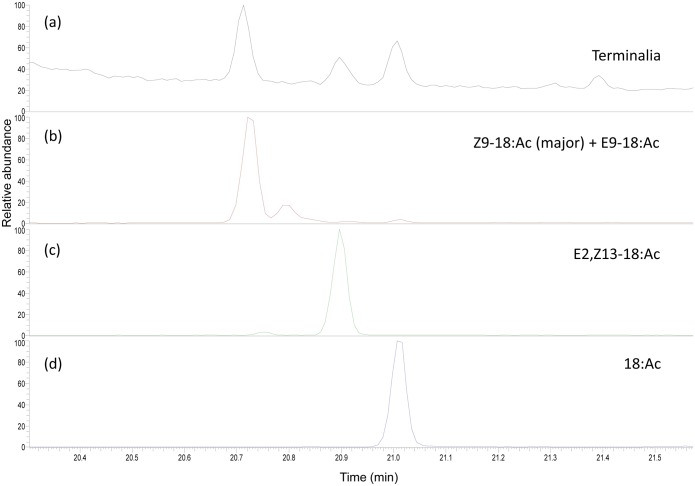
Chemical analysis of a terminalia gland extract of a *Paysandisia archon* male. (a) Partial GC-MS chromatogram of an extract of male terminalia of *P*. *archon* in comparison to the synthetic standards Z9-18:Ac (containing the E isomer as minor component) (b), E2,Z13-18:Ac (c), and 18:Ac (d).

A picture of the terminalia annular gland of a *P*. *archon* male is shown in Fig 4. Initial studies of the GC and GC-MS data of extracts of *P*. *archon* males terminalia (Fig 5) pointed out the presence of three acetates, namely 18:Ac, a possible C18 mono-unsaturated acetate and E2,Z13-18:Ac in relatively high amounts (18:Ac: 0.56±0.2 μg/male, possible monoene: 0.99±0.2 μg/male, E2,Z13-18:Ac: 0.40±0.1 μg/male, N = 6, 1–3 days old) (Fig 5). (For mass spectra of the natural acetates in comparison to those of synthetic materials see S2–S4 Figs).

**Fig 6:**
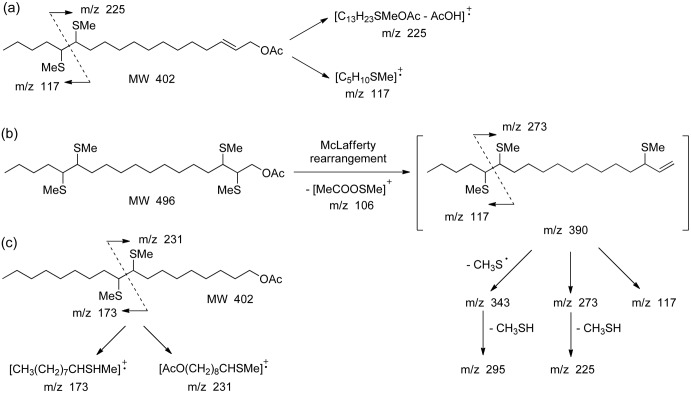
Localization of the double bonds of the diene acetate E2,Z13-18:Ac and monoene Z9-18:Ac present in a terminalia extract of a *Paysandisia archon* male. Assignment of the main ions in the mass spectrum of the addition compounds of one/two mol/es of DMDS to E2,Z13-18:Ac. Addition of the first mole takes place preferentially to the double bond at C-13 to give the mono-adduct of MW 402 (a). Addition of the second mole to the double bond at C-2 gives rise to the bis-adduct of MW 496 (see text) (b). Assignment of the diagnostic ions of the mass spectrum of the DMDS adduct of Z9-18:Ac (c).

None of the mono-unsaturated acetates prepared for this purpose (Z2-18:Ac, E2-18:Ac, and E3-18:Ac) nor any of the C18 mono-unsaturated acetates already available in our group (Z13-18:Ac and E13-18:Ac) matched the GC retention time of the first component both in non-polar (HP-5MS) and polar (DB-wax) columns. However, mass spectral evidence suggested that we were dealing with a mono-unsaturated C18 acetate. Therefore, to localize the double bond position and at the same time to confirm the presence of the E2,Z13 diene acetate, derivatization of the natural extract with DMDS was performed (for GC-MS chromatogram of a terminalia extract from a *P*. *archon* male after derivatization with DMDS see S5 Fig). The mass spectrum of the DMDS adduct of the monoene acetate (S6 Fig) showed diagnostic ions of m/z 173 and 231 which were assigned to the key fragments [CH_3_(CH_2_)_7_CHSMe]^+^ (fragment A^+^) and [AcO(CH_2_)_8_CHSMe]^+^ (fragment B^+^), respectively, resulting from the addition of DMDS to the double bond at C-9 (Fig 6). In this way, the monoene acetate was identified as Z9-18:Ac, which was confirmed by its GC behavior in comparison to that of a synthetic sample in the non-polar and polar columns.

For the diene acetate, both double bonds at C-2 and C-13 were amenable to react with one or two moles of DMDS giving rise to a mono- or bis-adduct, respectively (Fig 6). The first mole is preferentially added to the double bond at C-13 yielding the mono-adduct of MW 402, with diagnostic ions of m/z 117 and 225 corresponding to [CH_3_(CH_2_)_3_CHSMe]^+^(fragment A^+^), and to [CH_3_SCH(CH_2_)_9_CH = CHCH_2_OAc]^+^ (m/z 285 minus AcOH, fragment B^+^), respectively (Fig 6 and S7 Fig). The second mole of DMDS adds to the double bond at C-2 to give the bis-adduct of MW 496, which gives rise to the key fragment ion of m/z 390 through a McLafferty rearrangement with loss of methylthio acetate (CH_3_S-O-C = OCH_3_, M-106). This ion could probably be the parent ion of other diagnostic ions, such as those of m/z 343, 273, 295, 225, and 117 (Fig 6 and S8 Fig). In this way, the presence of E2,Z13-18:Ac was confirmed.

To locate a possible production site of the three acetates, a careful observation of the male terminalia led us to consider a ring-shaped gland as a potential site (Fig 4). Indeed, dissection and extracts analysis of the ventral part of the glands of 5-day-old males showed the presence of these compounds in noticeable amounts (18:Ac: 0.284±0.229 μg/male; Z9-18:Ac: 0.334±0.235 μg/male; E2,Z13-18:Ac: 0.255±0.139 μg/male, N = 5). These results were compared to those of the terminalia extracts and found no difference in the contents of the diene acetate (Student’s t-test, P = 0.094), although the difference was significant in the amounts of the other two acetates (Student’s t-test, P<0.05). This divergence could be due to the different age of the insects used (5 day-old males for the glands and 1–3 day-old for the terminalia) and/or to the fact that the gland extract was only from the ventral part of the gland (not from the whole gland). Interestingly, this type of ring-shaped epithelial exocrine gland is quite common in female moths, where it performs as a sex pheromone gland. In females, it is normally located in the ovipositor, between segments 8 and 9 of the abdomen, and consists of a thickened epithelial layer below the intersegmental cuticle. When the female moths call this glandular area is exposed [2, 18]. However, as far as we know, specialized scent or pheromone-producing gland structures in male lepidopterans adopt other formats, like those of coremata, androconia, Stobbe’s glands, brushes, alar organs, etc. (see [2] for review). Therefore, this newly discovered annular gland (of *ca*. 10–11 mm perimeter) located in the terminalia of *P*. *archon* males seems to be a new structure in male moths, making castniids even more peculiar lepidopterans. We name this new gland as CTG, standing for Castniids Terminalia Gland.

### Female ovipositors

**Fig 7:**
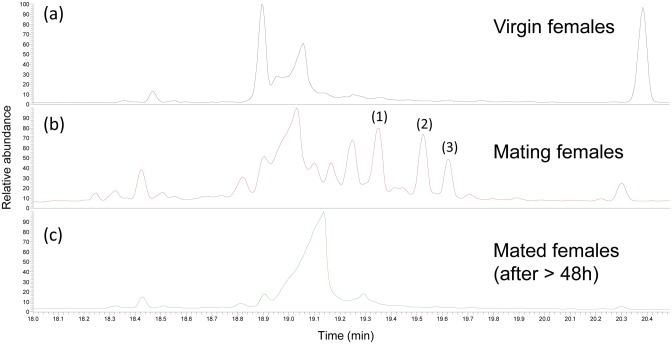
Analysis of ovipositor extracts of virgin, during mating, or mated *Paysandisia archon* females. Chromatograms of GC-MS analysis of extracts of the ovipositor of *P*. *archon* virgin females (a), during mating (b), and after mating (c). (1) = Z9-18:Ac, (2) = E2,Z13-18:Ac, (3) = 18:Ac. The other compounds shown in the figure are fatty acids (stearic, oleic and linoleic acids), long chain hydrocarbons and column phase artifacts.

Ovipositor extracts of virgin, during-mating, and mated females were analyzed to confirm the absence of female-produced pheromones, as already suggested [12–14] (Fig 7).

The extracts of virgin females (8 h after emergence) (N = 6 extracts of 12 ovipositors) and mated females (24–48 h after mating) (N = 4 extracts of 8 ovipositors) did not contain any detectable pheromone-like compound [19]. However, some ovipositor extracts of females *during mating* (N = 5 extracts of 11 ovipositors) were detected to contain the three acetates identified in male terminalia but only in very minor amounts, approximately 11.3 ng of E2,Z13-18:Ac, 8.7 ng of 18:Ac and 11.0 ng of Z9-18:Ac per ovipositor (ca. 20-30x lower than those detected in male glands). The compounds were only detected when two extracts were combined, concentrated to a very small volume and run in a single injection (Fig 7).

### Rubbing experiments

**Fig 8:**
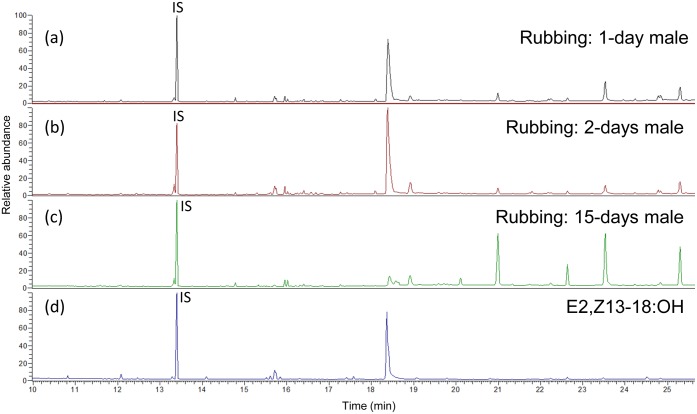
Gas chromatograms of *Chamaerops humilis* leaves rubbed by *Paysandisia archon* males. GC analysis of extracts of *C*. *humilis* leaves which had been rubbed with the midlegs of 1-day old (a), 2-day old (b), and 15-day old (c) *P*. *archon* males in comparison to the corresponding synthetic dienol E2,Z13-18:OH (d). IS = Z9-14:OH.

Palm (*C*. *humilis*) leaves that had been rubbed by *P*. *archon* males midlegs were extracted and analyzed (Fig 8). The leaves rubbed by 1- and 2-day-old males contained large amounts of E2,Z13-18:OH, an average of 8.35 μg/gr leaf (N = 2), whereas a 15-day-old male deposited only 0.99 μg/gr leaf (Fig 8) (for comparison of the mass spectrum of the dienol from an extract of leaves rubbed by *P*. *archon* males with that of a synthetic sample see S9 Fig). Palm leaves with no contact with the insects (control) lacked any trace of the chemical.

### EAG assays

Female antennae displayed small electrophysiological responses to the three acetates present in the male terminalia gland and to a mixture of them in a proportion similar to that found in the gland (Z9-18:Ac:18:Ac:E2,Z13-18:Ac 100:60:40) (S1 Table). The highest EAG responses were elicited by 10 μg of E2,Z13-18:Ac (0.118 ± 0.054 mV) and the ternary blend (0.114 ± 0.079 mV), whereas the saturated acetate and the mono-unsaturated compound elicited lower depolarizations (0.035 ± 0.025 mV and 0.069 ± 0.041 mV, respectively).

## Discussion

Castniid moths display a novel butterfly-like reproductive behavior based on the utilization of visual stimuli for mate location and an apparent lack of female-released pheromones [13]. A number of facts combined have led to the assumption that *P*. *archon* females have apparently lost their pheromone gland in an evolutionary context [4]. These facts include: (1) the territorial behavior of males; (2) the absence of compounds with pheromone-like activity in female ovipositors and other female parts [13, 20]; (3) *P*. *archon* females never adopt a typical calling position with the ovipositor extruded for a long period of time [4, 12]; (4) clubbed antennae with reduced sensillar area and no apparent sexual dimorphism [13, 21]; and (5) no evidence of pheromone gland tissues underneath the 8–9 intersegmental cuticle of the *P*. *archon* ovipositor [12, 13]. Against this assumption, Delle-Vedove et al. [15] claimed the identification of (E,Z)-2,13-octadecadienyl acetate as a female pheromone from ovipositor extracts of sexually mature females, and that “females exhibit calling behavior”. However, in our previous work, this compound failed to attract a single male when deployed in a filter paper alone or in a “dummy” cardboard [22]. In the present study, we have found remarkable amounts of this diene acetate in addition to Z9-18:Ac and 18:Ac in a ring-shaped gland of the male terminalia. These three acetates were EAG slightly active on female antennae, but not on males. The saturated acetate is a common pheromone component of a variety of species of moths and butterflies [19], among the latter *Tirumala formosa* (Godman), *Amauris echeria* (Stoll) and *A*. *niavius* (Linnaeus) (Nymphalidae: Danainae) [23]. Surprisingly, the mono-unsaturated acetate Z9-18:Ac has only been found as a very minor component of *Synanthedon haitangvora* Yang (Sesiidae: Sesiinae) pheromone [24]. The diene acetate, in turn, is a very common pheromone compound in moths of the families Cossidae, Tineidae and Sesiidae [19]. However, we have not found any pheromone-like compound in ovipositor extracts of *P*.*archon* virgin and mated females in detectable amounts. The other compounds shown in Fig 7 have been identified as fatty acids (stearic, oleic and linoleic acids), long chain hydrocarbons and column phase artifacts. Only occasionally, some ovipositor extracts of females *during mating* were detected to contain the three chemicals in very minor amounts (some ng per ovipositor) and in similar ratio to that found in male terminalia. We attribute this finding to a passive transfer of these compounds from the male to the female during mating. Our results suggest that these compounds are not used by mated females to avoid other courting males, and that they are simply absorbed by female terminal scales while in copula, disappearing soon afterwards. It is possible that the acetate found by Delle-Vedove et al. [14] from ovipositor extracts of *P*. *archon* and thought to be a female pheromone was in fact a male pheromone component which had been transferred during copula, as mentioned above. However, some questions arise. For instance, what is the real role of these male-produced acetates? Are they used as short-range aphrodisiacs for female acceptance as in danainae nymphalids? [25, 26]. Or, are they used as a sort of ‘lek’ pheromone [27, 28] to attract virgin females to male territories once the female approached the zone after the sight of palm trees?. Males are territorial but their areas can be quite close to each other conforming a ‘male’ zone.

The presence of Z,E-farnesal and E,E-farnesal in male wing extracts, which had been previously reported by us [13], was later put into question [17]. To shed light on this discrepancy, new extracts of forewings and hindwings of males of several ages were analyzed. We again detected both isomers of farnesal [13, 14] with the highest amounts being present in the median band of the hindwings of 48–53 h-old males. The amount of both isomers was significantly higher in the hindwings than in the forewings in all males regardless their age. Z,E-Farnesal was electrophysiologically active [13] but its biological significance in *P*. *archon* remains unknown. Farnesals, as such, have not been found as components of the larval host plants of *P*. *archon*, namely palm trees (Arecaceae), which do contain, however, the corresponding hydrocarbons Z,E- and E,E-farnesene [29]. These chemicals could be accumulated by the insect at the larval stage, biosynthetically converted into the corresponding alcohols and use these as pheromone precursors, as reported in other insects [30, 31]. Reports of farnesals as pheromone components of moths or butterflies are scarce. To our knowledge, only E,E-farnesal (as major) and its Z,E isomer were detected in extracts of male forewings of the rice moth *Corcyra cephalonica* (Stainton) (Pyralidae: Galleriinae) inducing walking attractancy on females [32]. Farnesal (no isomers cited) was also found in the mandibular glands secretion of the ant *Lasius fuliginosus* (Latreille) [33]. A homoterpene shorter analogue, citral (1:1 mixture of geranial and neral) has been recently found in male wings of the green-veined white butterfly *Pieris napi* (Linnaeus), and reported as an aphrodisiac pheromone released by a courting male to induce acceptance by the female [34]. Geranial and neral in different ratios have also been found as the main components of the scent scales of male butterflies *Pieris melete* Menetries and *P*. *napi japonica* Shirozu [35].

*P*. *archon* males also produce dienol E2,Z13-18:OH in large amounts, as previously reported [13, 17]. The chemical is mostly concentrated in the midlegs basitarsi and elicits significant electrophysiological responses on male and female antennae [17]. Production of the compound is maximum in 1 day-old males which correlates well with the fact that most individuals (males and females) are sexually mature 3 h after adult emergence [36]. This dienol has been previously found as component of the female sex pheromone of some species of Tineidae, Choreutidae, and, particularly, of various species of Sesiidae [19], the latter being closely related to Castniidae. The role of this compound in *P*. *archon* is not known. We hypothesize that it could act as an aggregation pheromone attracting individuals of both sexes and leading to the formation of *P*. *archon* groups near the signal's source, maybe using it as a cue for settlement. However, since males are territorial, its use as a ‘territorial’ or scent-marking pheromone to establish territorial boundaries in a way similar to that observed in mammals and other terrestrial vertebrates should not be ruled out. In addition, since *P*. *archon* males deposit this compound nearly exclusively on palm leaves, they might also use it as a host-marking pheromone.

An interesting “scratching behavior” by males was disclosed by Frérot et al. [15, 17]. The insect rubs its midlegs on the substratum “inducing take off and hovering flight in the female suggesting the release of chemicals contained by androconia”, as pointed by the authors. However, no experimental evidence was provided to support these claims. In this study, we report that extracts of leaves of the palm trees used as perching sites by *P*. *archon* males contained huge amounts of E2,Z13-18:OH when such leaves had been rubbed (or “scratched”) by males, whereas no trace of the compound was detected in non-rubbed leaves. To our knowledge, very few cases have been reported in which adult lepidopterans scratch living plants, and when this happened insects imbibe plant substances as precursors for the biosynthesis of pheromone components. For instance, several species of danainae butterflies, such as *Danaus chrysippus* (Linnaeus), *Amauris ochlea* (Boisduval) and *Tirumala petiverana* (Doubleday), have been seen scratching with their midlegs on leaves of *Heliotropium pectinatum* Vaupel (Boraginaceae) to get pyrrolizidine alkaloids [37] as precursors of pheromone components and/or as a defence mechanism against predators [38]. However, this behavior is quite different to that observed in *P*. *archon*. Males of this species do not scratch the leaves producing marks or wounds from where they can imbibe palm substances (they do not feed at all as adults); they simply rub them to deposit their dienol pheromone as mentioned above. The dienol seems to be spread on the leaves by the brush-like paronychia located at the distal tip of the midlegs. Therefore, we propose the term “rubbing” instead of “scratching” for this peculiar behavior of *P*. *archon*.

Altogether, and although the biological significance of the different compounds found in *P*. *archon* males is still far from being totally understood, our results suggest that the chemical communication of this day-flying castniid moth relies on males, which appear to bear all chemical burden. Indeed, so far all chemical, histological and ethological evidences point out towards the lack of any detectable female scent or pheromone in this insect, a situation similar to that found in female butterflies. This is likely to be extensible to at least many other species within the subfamily Castniinae, since many of the ethological and morphological traits mentioned for *P*. *archon* have also been observed in other species of this Neotropical group [11, 39, 40].

## Supporting information

S1 Fig *Paysandisia archon* male perching. on a leaf of the European fan palm *Chamaerops humilis*. (PDF)

S2 Fig Mass spectrum of 18:Ac from an extract of terminalia of a *P*. *archon* male in comparison to that of the synthetic material. (PDF)

S3 Fig Mass spectrum of E2,Z13-18:Ac from an extract of terminalia of a *P*. *archon* male in comparison to that of the synthetic material. (PDF)

S4 Fig Mass spectrum of Z9-18:Ac from an extract of terminalia of a *P*. *archon* male in comparison to that of the synthetic material. (PDF)

S5 Fig GC-MS chromatogram of a terminalia extract from a *P*. *archon* male after derivatization with DMDS. (PDF)

S6 Fig Mass spectrum of the DMDS adduct of Z9-18:Ac from a terminalia extract of *P*. *archon* males. (PDF)

S7 Fig Mass spectrum of the DMDS adduct of E2,Z13-18:Ac from a terminalia extract of *P*. *archon* males after addition on the double bond at C-13. (PDF)

S8 Fig Mass spectrum of the DMDS adduct of E2,Z13-18:Ac from a terminalia extract of *P*. *archon* males after addition on the two double bonds at C-2 and C-13. (PDF)

S9 Fig Mass spectrum of dienol E2,Z13-18:OH from an extract of leaves rubbed by *P*. *archon* males in comparison to that of the synthetic material. (PDF)

S1 Table EAG responses of *P*. *archon* females’ antennae to 18:Ac, Z9–18:Ac and E2,Z13-18:Ac and to the ternary blend in 60:100:40 ratio. (PDF)
