# Supplementary material for: Sexual communication in castniid moths: Males mark their territories and appear to bear all chemical burden
Source: PLoS One. 2017 Feb 8;12(2):e0171166. doi: 10.1371/journal.pone.0171166 (PMC5298307; doi:10.1371/journal.pone.0171166)
Supplement: S1 Fig — (PDF) [file pone.0171166.s001.pdf]

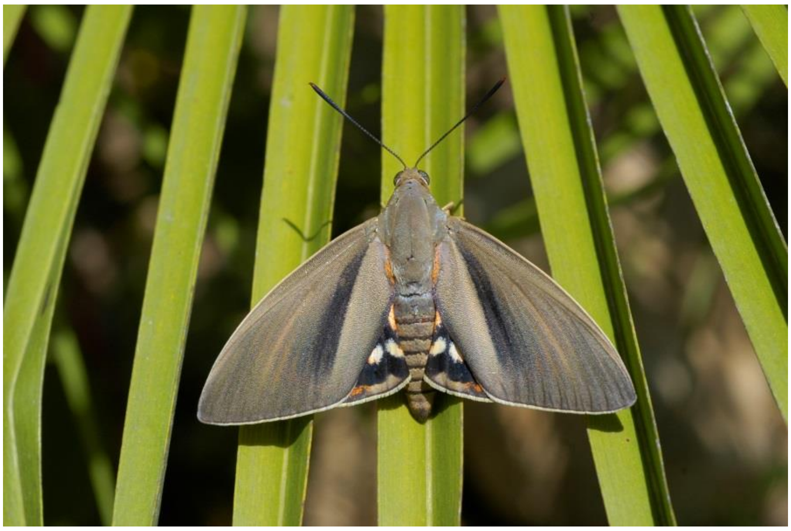

S1 Fig. ***Paysandisia archon*** **male perching on a leaf of the European fan palm** ***Chamaerops humilis*** (Photo V. Sarto i Monteys).
