# Supplementary material for: Sexual communication in castniid moths: Males mark their territories and appear to bear all chemical burden
Source: PLoS One. 2017 Feb 8;12(2):e0171166. doi: 10.1371/journal.pone.0171166 (PMC5298307; doi:10.1371/journal.pone.0171166)
Supplement: S2 Fig — (PDF) [file pone.0171166.s002.pdf]

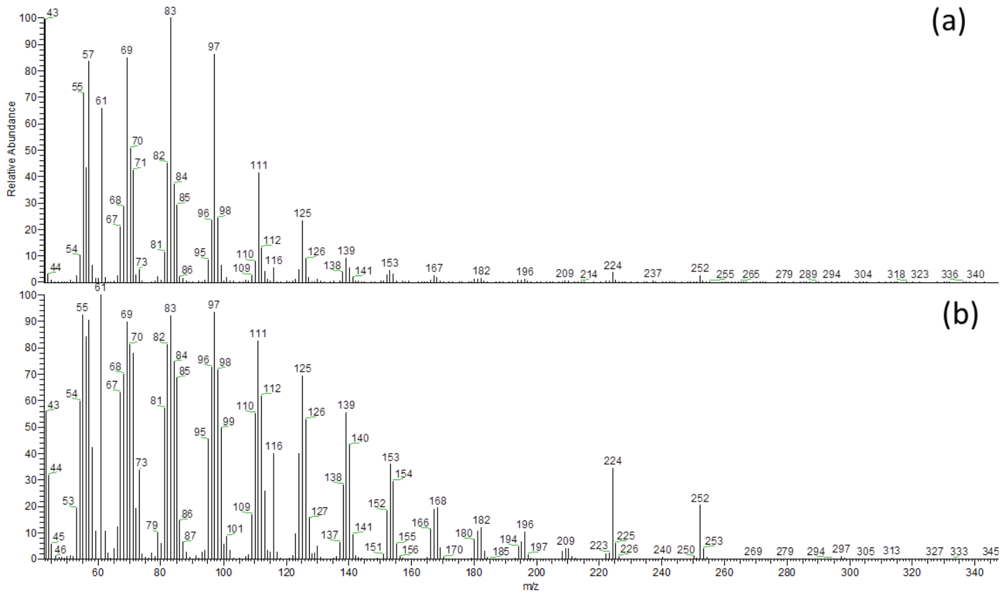

S2 Fig. **Mass spectrum of 18:Ac from an extract of terminalia of a *P. archon* male (a) in comparison to that of the synthetic material (b)**
