# Supplementary material for: Sexual communication in castniid moths: Males mark their territories and appear to bear all chemical burden
Source: PLoS One. 2017 Feb 8;12(2):e0171166. doi: 10.1371/journal.pone.0171166 (PMC5298307; doi:10.1371/journal.pone.0171166)
Supplement: S3 Fig — (PDF) [file pone.0171166.s003.pdf]

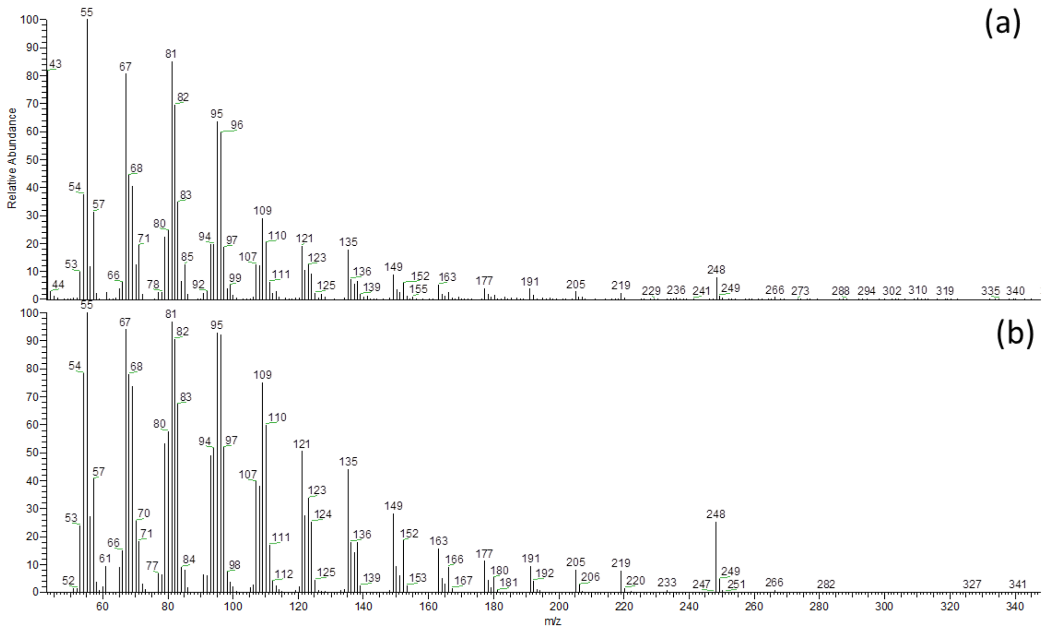


**S3 Fig**. **Mass spectrum of E2,Z13-18:Ac from an extract of terminalia of a *P. archon* male (a) in comparison to that of the synthetic material (b).**
