# Supplementary material for: Sexual communication in castniid moths: Males mark their territories and appear to bear all chemical burden
Source: PLoS One. 2017 Feb 8;12(2):e0171166. doi: 10.1371/journal.pone.0171166 (PMC5298307; doi:10.1371/journal.pone.0171166)
Supplement: S5 Fig — (PDF) [file pone.0171166.s005.pdf]

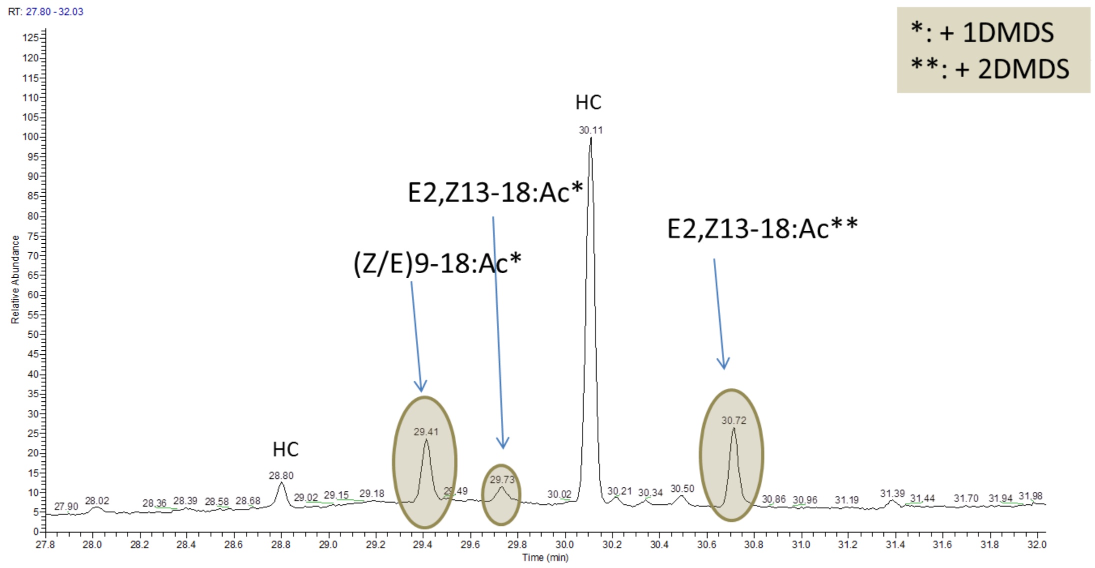

S5 Fig. **GC-MS chromatogram of a terminalia extract from a *P. archon* male after derivatization with DMDS.** Peaks of retention time 29.41 and 29.73 min correspond to the adduct resulting from the addition of one mole of DMDS to the double bond at C-9 of Z9-18:Ac and C-13 of E2,Z13-18:Ac, respectively. Peak of retention time 30.72 min corresponds to the adduct resulting from the addition of one mole of DMDS to both double bonds at C-2 and C-13 of E2,Z13-18:Ac. HC: long chain hydrocarbon
