# Supplementary material for: Sexual communication in castniid moths: Males mark their territories and appear to bear all chemical burden
Source: PLoS One. 2017 Feb 8;12(2):e0171166. doi: 10.1371/journal.pone.0171166 (PMC5298307; doi:10.1371/journal.pone.0171166)
Supplement: S6 Fig — (PDF) [file pone.0171166.s006.pdf]

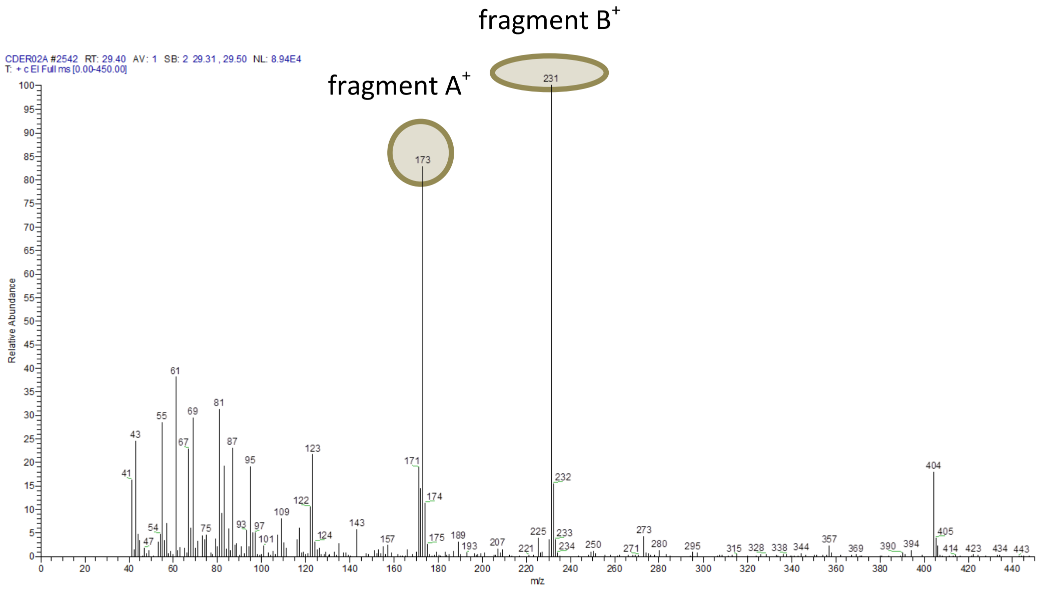

S6 Fig. **Mass spectrum of the DMDS adduct of Z9-18:Ac from a terminalia extract of *P. archon* males**. Diagnostic ions of m/z 173 and 231 correspond to the key fragments $[CH_3(CH_2)_7CHSMe]^+$ (fragment A^+) and $[AcO(CH_2)_8CHSMe]^+$ (fragment B^+), respectively, resulting form the addition of one mol of DMDS to the double bond of Z9-18:Ac.
