# Supplementary material for: Sexual communication in castniid moths: Males mark their territories and appear to bear all chemical burden
Source: PLoS One. 2017 Feb 8;12(2):e0171166. doi: 10.1371/journal.pone.0171166 (PMC5298307; doi:10.1371/journal.pone.0171166)
Supplement: S7 Fig — (PDF) [file pone.0171166.s007.pdf]

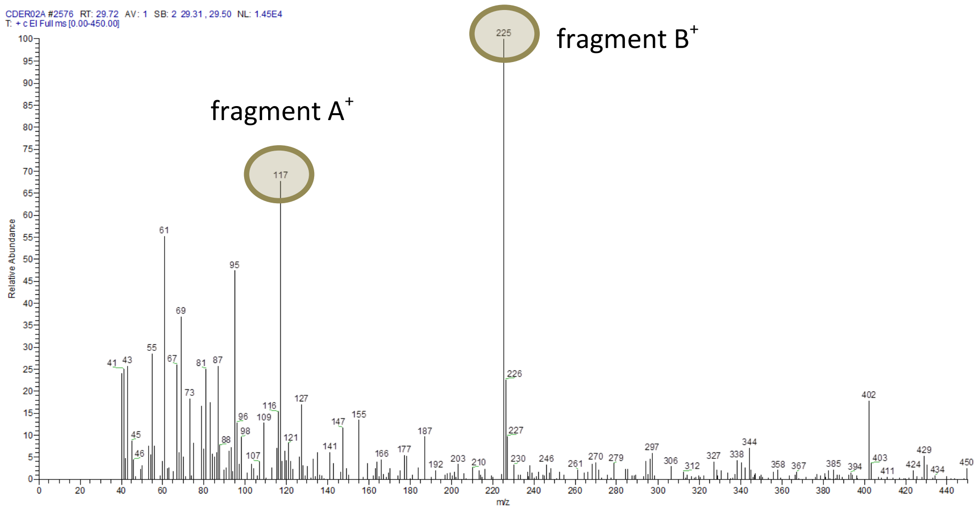

S7 Fig. Mass spectrum of the DMDS adduct of E2,Z13-18:Ac from a terminalia extract of *P. archon* males after addition on the double bond at C-13. Diagnostic ions of m/z 117 and 225 correspond to $[CH_3(CH_2)_3CHSMe]^+$ (fragment A^+), and $[CH_3SCH(CH_2)_9CH{=}CHCH_2OAc]^+$ (m/z 285) minus AcOH (fragment B^+), respectively.
