# Supplementary material for: Sexual communication in castniid moths: Males mark their territories and appear to bear all chemical burden
Source: PLoS One. 2017 Feb 8;12(2):e0171166. doi: 10.1371/journal.pone.0171166 (PMC5298307; doi:10.1371/journal.pone.0171166)
Supplement: S8 Fig — (PDF) [file pone.0171166.s008.pdf]

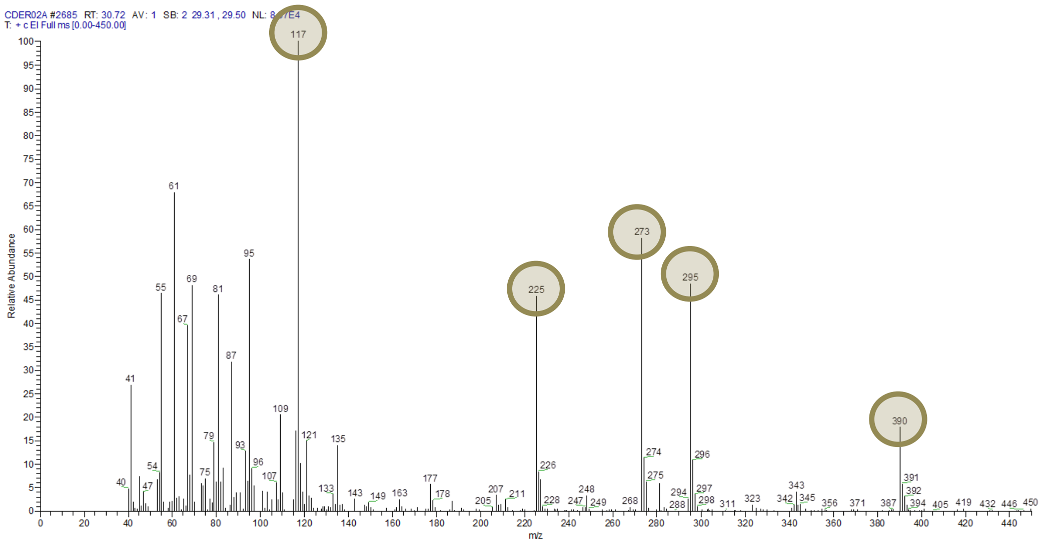

S8 Fig. **Mass spectrum of the DMDS adduct of E2,Z13-18:Ac from a terminalia extract of *P. archon* males after addition on the two double bonds at C-2 and C-13**. For assignment of diagnostic ions of m/z 117, 225, 273, 295 and 390 see main text.
