# Supplementary material for: Sexual communication in castniid moths: Males mark their territories and appear to bear all chemical burden
Source: PLoS One. 2017 Feb 8;12(2):e0171166. doi: 10.1371/journal.pone.0171166 (PMC5298307; doi:10.1371/journal.pone.0171166)
Supplement: S9 Fig — (PDF) [file pone.0171166.s009.pdf]

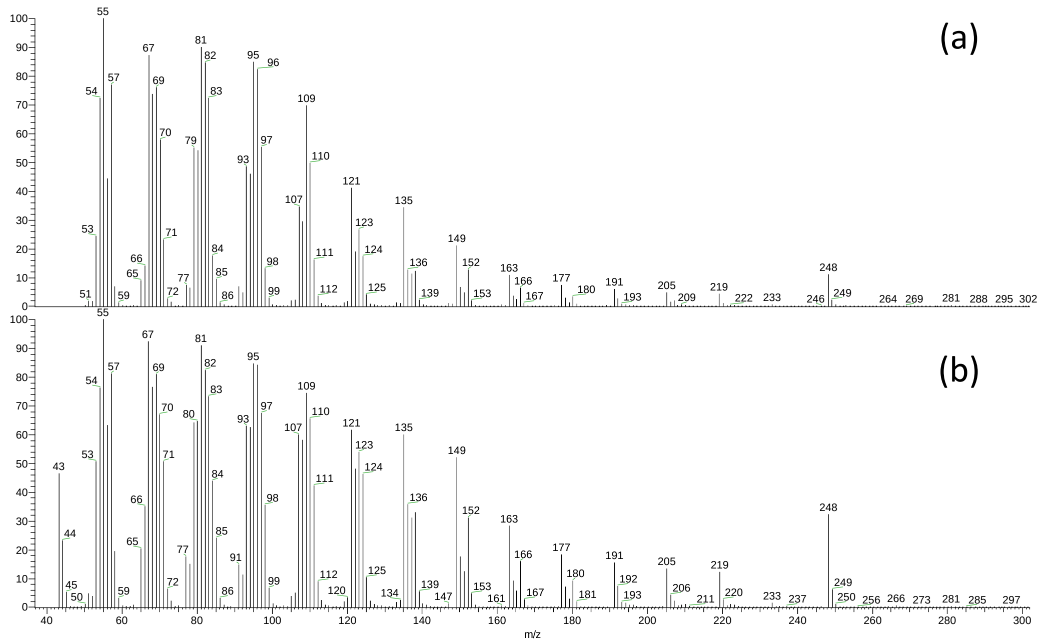

S9 Fig. **Mass spectrum of E2,Z13-18:OH from an extract of leaves rubbed by *P. archon* males (a) and synthetic material (b).**
