# Supplementary material for: Sexual communication in castniid moths: Males mark their territories and appear to bear all chemical burden
Source: PLoS One. 2017 Feb 8;12(2):e0171166. doi: 10.1371/journal.pone.0171166 (PMC5298307; doi:10.1371/journal.pone.0171166)
Supplement: S1 Table — (PDF) [file pone.0171166.s010.pdf]

S1 Table. **EAG responses (mV) of antennae of *P. archon* females (N=3) to different doses of 18:Ac, Z9-18:Ac and E2,Z13-18:Ac and to the ternary blend in 60:100:40 ratio, respectively.**

	**18:Ac**	**Z9-18:Ac**	**E2,Z13-18:Ac**	**Ternary blend**
1 µg	NR[1]	NR[1]	NR[1]	0.072±0.046
10µg	0.035±0.025	0.069±0.041	0.118±0.054	0.114±0.079

[1]No response.
